# Ocular changes during hemodialysis in patients with end-stage renal disease

**DOI:** 10.1186/s12886-018-0885-0

**Published:** 2018-08-23

**Authors:** Hejun Chen, Xi Zhang, Xi Shen

**Affiliations:** 1 Ruijin Hospital Affiliated to Shanghai Jiao Tong University School of Medicine, No. 197 Rui Jin Er Road, Shanghai, 200025 China; 2 Xinhua Hospital Affiliated to Shanghai Jiao Tong University School of Medicine, No.1665 Kongjiang Road, Shanghai, 200092 China

**Keywords:** End-stage renal disease, Hemodialysis, Ocular changes

## Abstract

**Background:**

To explore ocular changes during hemodialysis (HD) in chronic renal failure patients and to determine the effects of different causes of renal failure during HD.

**Methods:**

A total of 90 eyes from 45 end-stage renal disease (ESRD) patients undergoing HD were evaluated in this study. All ophthalmological examinations were conducted within 1 h before and after a single HD session. The HD patients were divided into primary kidney disease (KD), hypertensive KD, diabetic KD (DM-KD) and unknown etiology subgroups according to the primary etiology of renal failure. The statistics of 38 eyes from 19 healthy people were set as normal control.

**Results:**

Tear break-up time (TBUT) (*P* = 0.020), Schirmer’s I test results (*P* = 0.030), anterior chamber depth (ACD) (*P* = 0.006), lens thickness (LT) (*P* < 0.001) and choroidal thickness (CHT) (P < 0.001)decreased significantly after a single HD. The retinal nerve fiber layer (RNFL) thickness and average retinal thickness (RT) increased after HD, especially in the nasal inner macula (NIM) subfield (*P* < 0.001), the inferior inner macula (IIM) subfield (*P* = 0.004) and the superior outer macula (SOM) subfield (*P* = 0.012). TBUT, Schirmer’s I test, IOP, RT, and CHT were correlated with one or more parameters. All ESRD patients regardless of etiology had the same trend for most parameters during HD, with the exception of the logMAR of BCVA, central corneal thickness, RNFL thickness and CHT.

**Conclusions:**

HD may affect a range of ocular parameters in ESRD patients. Dry eye parameters, RT and CHT exhibited the most obvious changes. Different etiologies tended to have similar trends in ocular parameter changes during HD.

## Background

End-stage renal disease (ESRD), with a glomerular filtration rate lower than 15 ml/ (min*1.73 m^2^), is the 5th stage of disease and the final outcome of disease progression in chronic kidney disease (CKD) patients. At this stage, a variety of clinical manifestations, such as hypertension, anemia, and edema, and metabolic and endocrine disorders can occur; thus, renal replacement therapy, such as hemodialysis (HD), is needed to remove excess water and metabolic wastes from the extracorporeal blood and to maintain the electrolyte and acid-base balance [1].

The negative impact of CKD on the patient’s eye is complex and diverse. Studies have shown that HD, as a relief and treatment of CKD, can improve certain ocular symptoms in ESRD patients. It has been reported that best corrected visual acuity (BCVA) improves after a single HD session, and patients with diabetes tend to have more obvious improvements [2, 3]. Other researchers have reported that HD can relieve macular edema in patients with kidney failure caused by diabetes [4]. However, in most cases, the negative impact of hemodialysis on the eye in CKD patients seems to be far beyond its positive impact. Aktas et al. [5] found aggravation of dry eye syndrome after a single session of HD. Moreover, it has been observed since the early sixties that HD can change the level of intraocular pressure (IOP). Different studies have shown IOP to increase [6–8], decrease [9] or remain unchanged [10]. The effects of HD on the posterior pole include changes in retinal thickness, retinal nerve fiber layer (RNFL) thickness, and choroidal thickness. Significant differences in these parameters have been reported in some studies, although others hold different views [11–13].

Since most of the effects of HD on ophthalmological parameters remain unclear and because no studies have reported the effects of HD on different etiologies, we conducted this study including a total of 45 patients with ESRD who underwent HD and analyzed the changes of both laboratory test and ocular parameters after HD to investigate the effects of the hemodialysis on the eye.

## Methods

### Subjects

CKD stage 5 patients undergoing hemodialysis treatment for at least 3 months at the Blood Purification Center of Ruijin Hospital affiliated with Shanghai Jiao Tong University from February 2014 to October 2014 were enrolled in this study. Hemodialysis was performed 3 times a week, each lasting 3–5 h.

The inclusion criteria were visual acuity over 20/200 as well as Oculus Pentacam® anterior segment analyzer (Oculus Inc., Wetzlar, Germany) results and OCT reports of acceptable quality. The exclusion criteria were a history of surgical or laser-based operations to the eye, other ocular diseases such as corneal scarring, uveitis, macular holes, and other conditions, or a history of renal replacement therapy, including peritoneal dialysis and kidney transplantation.

The hemodialysis group was divided into primary kidney disease (KD), hypertensive KD and diabetic KD (DM-KD) subgroups strictly according to the initial etiology of renal insufficiency. When patients failed to provide reliable supporting materials or when two or more etiologies were suspected, the patients were included in the etiology unknown subgroup. Nineteen healthy people without HD history were set as normal control.

This study adhered to the Declaration of Helsinki and was approved by the institutional review board of Shanghai Ruijin Hospital. Informed consent was obtained from the subjects after verbal and written explanations of the nature and possible consequences of the study were provided.

### Protocol

Blood reports, including urea, creatinine, uric acid, serum electrolytes (Na, K, Ca, P, Mg), parathyroid hormone (PTH), and fasting blood glucose levels, were collected before hemodialysis.

Blood pressure and detailed ophthalmological examinations, including spherical and cylinder powers, BCVA, IOP, dry eye analysis, corneal endothelial measurements, central corneal thickness (CT), anterior chamber depth (ACD), lens measurements, retinal thickness (RT) around the fovea, RNFL thickness, and choroidal thickness (CHT), were performed. BCVA was examined using a standard vision chart, and the logarithmic minimum angle of resolution (logMAR) was recorded. Refractive parameters were measured by a full auto ref-keratometer (Canon, Japan). IOP was measured by non-contact tonometer (NCT) (Canon, Japan). Dry eye syndrome was estimated by the tear break-up time (TBUT) and Schirmer’s I test. Endothelial cell density (ECD), the average endothelial cell size (ECS), and the endothelial cell size variation coefficient (ECSCV) were obtained using a Tomey EM-3000 non-contact specular microscope corneal endothelial cell counter. The CT, ACD and lens thickness (LT) were automatically calculated using a Pentacam® anterior segment analyzer (Oculus Inc., Wetzlar, Germany). The RT and RNFL thickness was measured using Cirrus HD-OCT (Carl Zeiss Meditec, Inc., Dublin CA, United States) under the Macular Cube 512 × 128 mode. The RT was defined as the average thickness of the 6 mm × 6 mm scan. The nine subfields of the RT map were measured separately. The inner, intermediate, and outer rings had radii of 1 mm, 3 mm, and 6 mm, respectively. The average thickness within the inner ring was defined as the central foveal subfield (CSF) thickness [14]. The average CHT was measured in the EDI mode of OCT. The subfoveal, nasal and temporal choroidal thicknesses were each measured 1 mm and 2 mm away from the center of the macula, averaged, and recorded as the average thickness of the choroid.

All examinations were measured within an hour before and after a single session of hemodialysis.

### Statistical analysis

Paired *t*-tests were used for indexes measured before and after HD if homogeneity of variance was verified. If homogeneity of variance was not verified, then a non-parametric test was used. Multiple linear regression analyses were conducted to identify correlations between parameters. Then, single factor analysis of variance (one-way ANOVA) and least-significant difference (LSD) tests were performed to determine parameters with significant changes during HD among subgroups according to their etiological classification. Lastly, one-way ANOVA was used to compare parameters with significant changes. A box plot was drawn, and Tukey’s test was used to isolate data with large deviations. The *P*-values, Estimated Marginal Means and their standard errors (SEs) were calculated by General Estimating Equations (which automatically take into account paired eye data from the same subject) after adjusting for age, sex, eyes, measurement times, HD duration, and primary diseases.

SPSS22.0 software was used to analyze the data. Statistical significance was considered when *P* < 0.05. All data are presented as the means ± standard deviation (means ± SD). All values are expressed as the means ± SD.

## Results

### Demographic characteristics of the HD patients and healthy controls

A total of 90 eyes from both sides of 45 HD patients were included in the study, including 27 pairs of male eyes (60.00%) and 18 pairs of female eyes (40.00%). The average age was 57.48 ± 13.57 years. The mean dialysis time was 70.09 ± 58.03 months. The patients were divided into 3 subgroups: the primary KD subgroup, the hypertensive KD subgroup and the DM-KD subgroup. No differences were found in age (*P* = 0.639) or dialysis time (*P* = 0.270) among the 3 subgroups.

Thirty-eight eyes from 19 healthy people were set as normal control. The blood pressure (both systolic and diastolic), urea, creatinine, uric acid, Na, K, P, Mg, PTH, fasting plasma glucose were found statistically different from the pre-HD ESRD patients. However, most of the ocular parameters, except for Spherical power and a few part of the retinal thickness showed no statistical difference (Table 1).

**Table 1:** Demographic characteristics of the hemodialysis group (pre HD) and the control group: comparison of basic situation and baseline data (Mean ± SD)

Pre HD	HD Group	Control Group	*P* value
Male [eyes (percentage)]	54 (60.00%)	60 (63.83%)	0.593
Female [eyes (percentage)]	36 (40.00%)	34 (36.17%)	
Age (year)	57.78 ± 13.57	54.90 ± 17.60	0.239
Systolic pressure (mmHg)	147.14 ± 22.43	133.65 ± 18.64	0.016*
Diastolic pressure (mmHg)	83.75 ± 16.03	75.28 ± 11.79	0.022*
Urea (mmol/L)	24.330 ± 7.853	5.275 ± 0.936	< 0.001**
Creatinine (umol/L)	891.10 ± 70.78	77.00 ± 20.38	< 0.001**
Uric acid (umol/L)	427.50 ± 79.78	367.88 ± 85.441	0.012*
Na (mmol/L)	138.20 ± 2.64	142.00 ± 2.45	< 0.001**
K (mmol/L)	4.860 ± 0.920	4.191 ± 0.217	< 0.001**
Ca (mmol/L)	2.279 ± 0.206	2.283 ± 0.070	0.908
P (mmol/L)	1.849 ± 0.542	1.160 ± 0.180	< 0.001**
Mg (mmol/L)	1.050 ± 0.129	0.873 ± 0.035	< 0.001**
PTH (ng/L)	479.70 ± 419.20	40.30 ± 12.45	< 0.001**
Fasting plasma glucose (mmol/L)	7.725 ± 3.356	5.433 ± 0.695	< 0.001**
Spherical power (D)	0.088 ± 0.508	−2.4011 ± 3.7786	0.012*
Cylinder power (D)	−0.857 ± 0.138	−0.6711 ± 0.6071	0.070
logMAR of BCVA	0.100 ± 0.0282	− 0.0205 ± 0.2344	0.345
TBUT(s)	6.957 ± 0.861	9.171 ± 5.801	0.949
Schirmer’s I test (mm)	12.014 ± 2.020	16.934 ± 9.617	0.309
IOP (mmHg)	12.855 ± 0.420	15.01 ± 3.69	0.073
ACD (mm)	2.642 ± 0.073	2.783 ± 0.481	0.082
CT (μm)	575.18 ± 5.859	549.11 ± 38.67	0.650
ECD (cell/mm^2^)	2758.47 ± 35.494	2707.46 ± 194.91	0.055
ECS (μm^2^)	363.16 ± 6.577	371.34 ± 29.27	0.132
ECSCV	40.717 ± 1.573	40.88 ± 7.35	0.381
LT (mm)	4.146 ± 0.064	3.979 ± 0.460	0.120
CSF (μm)	244.36 ± 4.464	249.68 ± 32.75	0.080
SIM (μm)	312.22 ± 2.912	323.45 ± 22.49	0.350
NIM (μm)	310.28 ± 2.904	322.13 ± 21.33	0.220
IIM (μm)	311.14 ± 3.990	316.29 ± 17.85	0.022*
TIM (μm)	302.88 ± 4.233	310.79 ± 19.39	0.030*
SOM (μm)	276.82 ± 3.915	280.42 ± 16.98	0.475
NOM (μm)	289.25 ± 2.368	294.61 ± 19.35	0.428
IOM (μm)	267.27 ± 5.037	266.45 ± 19.31	0.204
TOM (μm)	259.62 ± 5.432	262.37 ± 20.73	0.290
Average RT (μm)	273.40 ± 3.302	276.39 ± 17.37	0.164
RNFL thickness (μm)	90.65 ± 1.829	96.24 ± 23.06	0.669
CHT (μm)	289.55 ± 11.385	232.87 ± 65.23	0.302

*PTH* Parathyroid hormone, *TBUT* Tear break-up time, *logMAR* Logarithmic minimum angle of resolution, *BCVA* Best corrected visual acuity, *IOP* Intraocular pressure, *ACD* Anterior chamber depth, *LT* Lens thickness, *CT* Corneal thickness, *ECD* Endothelial cell density, *ECS* Endothelial cell size, *ECSCV* Endothelial cell size variation coefficient, *CSF* Central subfield, *SIM* Superior inner macula, *NIM* Nasal inner macula, *IIM* Inferior inner macula, *TIM* Temporal inner macula, *SOM* Superior outer macula, *NOM* Nasal outer macula, *IOM* Inferior outer macula, *TOM* Temporal outer macula, *MV* Macular volume, *RNFL* Retinal nerve fiber layer, *CHT* Choroidal thickness, *: *P* <  0.05; **: *P*<0.01

### A comparison of blood pressure during HD

The average systolic pressure (SP) before HD was 147.14 ± 22.43 mmHg, which increased to 136.09 ± 24.37 mmHg after HD (*P* = 0.001). The mean diastolic blood pressure (DP) decreased from 83.75 ± 16.03 mmHg to 76.48 ± 13.47 mmHg (*P* = 0.006).

### Conjunctival and corneal calcification

**Fig. 1:**
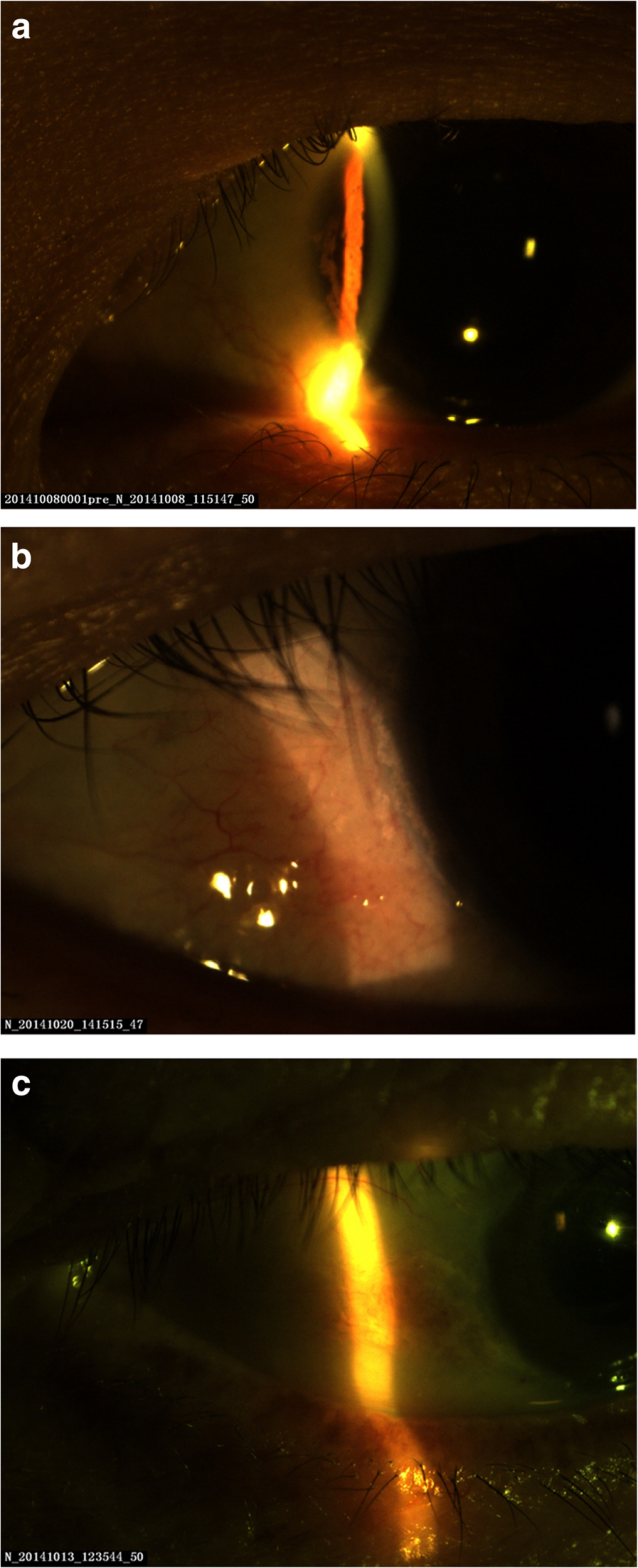
Conjunctival and corneal calcification. **a** In patient A, who had a 7-year HD history, a gray, line-shaped calcium deposit could be seen on the cornea. **b** In patient B, who had a 3-year HD history, white dot- and line-shaped calcium deposits were observed at the limbus. **c** In patient C, who had a 6-year HD history, white, block-shaped conjunctival calcium deposits were observed

Visible calcium deposits were spotted on the cornea or conjunctiva in 22 (48.89%) of the 45 hemodialysis patients. These deposits were located in the nasal and/or temporal side of the cornea, conjunctiva and limbus. They were either white or gray and were point-, line- or block-shaped (Fig. 1).

### A comparison of the ocular surface, BCVA, and refractive parameters during HD

After HD, the TBUT decreased from 6.957 ± 0.861 s to 5.205 ± 0.670 s (*P* = 0.020), and the Schirmer’s I test results decreased from 12.014 ± 2.020 mm to 9.964 ± 1.912 mm (*P* = 0.030). However, no significant change was observed in the BCVA (*P* = 0.880), spherical power (*P* = 0.442) or cylinder power (*P* = 0.937) measurements during HD (Table 2).

**Table 2:** Comparison of the ocular surface, BCVA, and refractive parameters during HD

HD group parameters	Pre (Estimated Marginal Mean ± SE)	Post (Estimated Marginal Mean ± SE)	*P* value
TBUT (s)	6.957 ± 0.861	5.205 ± 0.670	0.020*
Schirmer’s I test (mm)	12.014 ± 2.020	9.964 ± 1.912	0.030*
logMAR of BCVA	0.100 ± 0.0282	0.099 ± 0.027	0.880
Spherical power (D)	0.088 ± 0.508	0.032 ± 0.498	0.442
Cylinder power (D)	−0.857 ± 0.138	− 0.863 ± 0.148	0.937

*TBUT* Tear break-up time, *logMAR* Logarithmic minimum angle of resolution, *BCVA* Best corrected visual acuity, *: *P* < 0.05

The *P*-values, Estimated Marginal Means and their standard errors (SEs) were calculated by General Estimating Equations after adjusting for age, sex, eyes, measurement times, HD duration, and primary diseases

A significant positive correlation was found between the TBUT change within each HD session and the duration of all HD treatments (*B* = 0.040, *T* = 3.670, *P* = 0.001). Meanwhile, the change in the Schirmer’s I test results (*B* = − 0.566, *T* = − 5.121, *P* < 0.001) and blood urea levels (*B* = − 0.271, *T* = − 2.179, *P* = 0.036) were negatively correlated.

### Comparison of the IOP and anterior segment parameters during HD

The ACD decreased from 2.642 ± 0.073 mm to 2.613 ± 0.077 mm after HD (*P* = 0.006). The mean LT dropped from 4.153 ± 0.413 mm to 4.056 ± 0.389 mm (*P* < 0.001). No significant differences were observed in IOP (*P* = 0.113), CT (*P* = 0.643), ECD (*P* = 0.807), ECS (*P* = 0.164), or ECSCV (*P* = 0.348) during HD (Table 3).

**Table 3:** Comparison of the IOP and anterior segment parameters during HD

HD group parameters	Pre (Estimated Marginal Mean ± SE)	Post (Estimated Marginal Mean ± SE)	*P* value
IOP (mmHg)	12.855 ± 0.420	12.292 ± 0.476	0.113
ACD (mm)	2.642 ± 0.073	2.613 ± 0.077	0.006*
LT (mm)	4.146 ± 0.064	4.049 ± 0.063	< 0.0001*
CT (μm)	575.18 ± 5.859	579.62 ± 5.427	0.643
ECD (cells/mm^2^)	2758.47 ± 35.494	2766.55 ± 41.074	0.807
ECS (μm^2^)	363.16 ± 6.577	359.36 ± 5.120	0.164
ECSCV	40.717 ± 1.573	40.073 ± 1.306	0.348

*IOP* Intraocular pressure, *ACD* Anterior chamber depth, *LT* Lens thickness, *CT* Corneal thickness, *ECD* Endothelial cell density, *ECS* Endothelial cell size, *ECSCV* Endothelial cell size variation coefficient, *: *P* < 0.05

The *P*-values, Estimated Marginal Means and their SEs were calculated by General Estimating Equations after adjusting for age, sex, eyes, measurement times, HD duration, and primary diseases

We found a negative correlation between changes in IOP during HD and diastolic pressure (*B* = − 0.068, *T* = − 3.606, *P* = 0.001).

### A comparison of the posterior segment parameters during HD

The average retinal thickness increased from 273.40 ± 3.302 μm to 275.60 ± 3.180 μm (*P* = 0.071), especially in the nasal inner macula (NIM) subfield (*P* < 0.001), the inferior inner macula (IIM) subfield (*P* = 0.004) and the superior outer macula (SOM) subfield (*P* = 0.012). The remaining subfields had no significant differences. The RNFL thickness increased from 90.65 ± 1.829 μm to 93.18 ± 1.974 μm (*P* = 0.001). The CHT decreased from 289.55 ± 11.385 μm to 254.134 ± 11.46 μm (*P* < 0.001) (Table 4).

**Table 4:** Comparison of the posterior segment parameters during HD

HD group parameters	Pre (Estimated Marginal Mean ± SE)	Post (Estimated Marginal Mean ± SE)	*P* value
Average RT (μm)	273.40 ± 3.302	275.60 ± 3.180	0.071
CSF (μm)	244.36 ± 4.464	246.56 ± 4.575	0.308
SIM (μm)	312.22 ± 2.912	312.43 ± 2.717	0.895
NIM (μm)	310.28 ± 2.904	313.21 ± 3.037	< 0.001*
IIM (μm)	311.14 ± 3.990	313.53 ± 4.090	0.004*
TIM (μm)	302.88 ± 4.233	304.58 ± 3.889	0.150
SOM (μm)	276.82 ± 3.915	282.49 ± 4.127	0.012*
NOM (μm)	289.25 ± 2.368	291.42 ± 2.636	0.064
IOM (μm)	267.27 ± 5.037	266.69 ± 4.816	0.570
TOM (μm)	259.62 ± 5.432	261.21 ± 4.707	0.409
RNFL thickness (μm)	90.65 ± 1.829	93.18 ± 1.974	0.001*
CHT (μm)	289.55 ± 11.385	254.134 ± 11.46	< 0.001*

*CSF* Central subfield, *SIM* Superior inner macula, *NIM* Nasal inner macula, *IIM* Inferior inner macula, *TIM* Temporal inner macula, *SOM* Superior outer macula, *NOM* Nasal outer macula, *IOM* Inferior outer macula, *TOM* Temporal outer macula, *MV* Macular volume, *RNFL* Retinal nerve fiber layer, *CHT* Choroidal thickness, *: *P* < 0.05

The *P*-values, Estimated Marginal Means and their SEs were calculated by General Estimating Equations after adjusting for age, sex, eyes, measurement times, HD duration, and primary diseases

A positive correlation was found between the average RT change and the central corneal thickness (*B* = 0.130, *T* = 5.127, *P* < 0.001) and the potassium level (*B* = 3.950, *T* = 2.650, *P* = 0.012) before HD. The choroidal thickness change was positively related to TBUT (*B* = 3.120, *T* = 3.637, P = 0.001) and was negatively correlated with sodium level (B = − 4.163, *T* = − 2.241, *P* = 0.031) and ACD (B = − 30.190, *T* = − 2.356, *P* = 0.024) before HD.

### Comparison of parameters between subgroups according to the original cause of HD

Both eyes of all HD patients were divided into 4 subgroups according to the etiology of ESRD: the primary kidney disease (KD) subgroup (*n* = 27), the hypertensive KD subgroup (*n* = 9), the diabetic mellitus KD (DM-KD) subgroup (*n* = 6) and the etiology unknown subgroup (*n* = 3). The 3 etiology unknown patients were not included in the following analysis (Table 5).

**Table 5:** Demographic characteristics of the 3 etiological ESRD subgroups

Characteristics	Primary KD *n* = 45
Number of eyes (eyes)	90
Men, n (%)	27 (60.0%)
Age (years)	57.78 ± 13.57
HD duration (months)	70.09 ± 58.03
Primary Diseases, n (%)	27 (60.0)
Hypertensive KD, n (%)	9 (20.0)
DM-KD, n (%)	6 (13.3)

*KD* Kidney disease, *DM* Diabetes mellitus

**Fig. 2:**
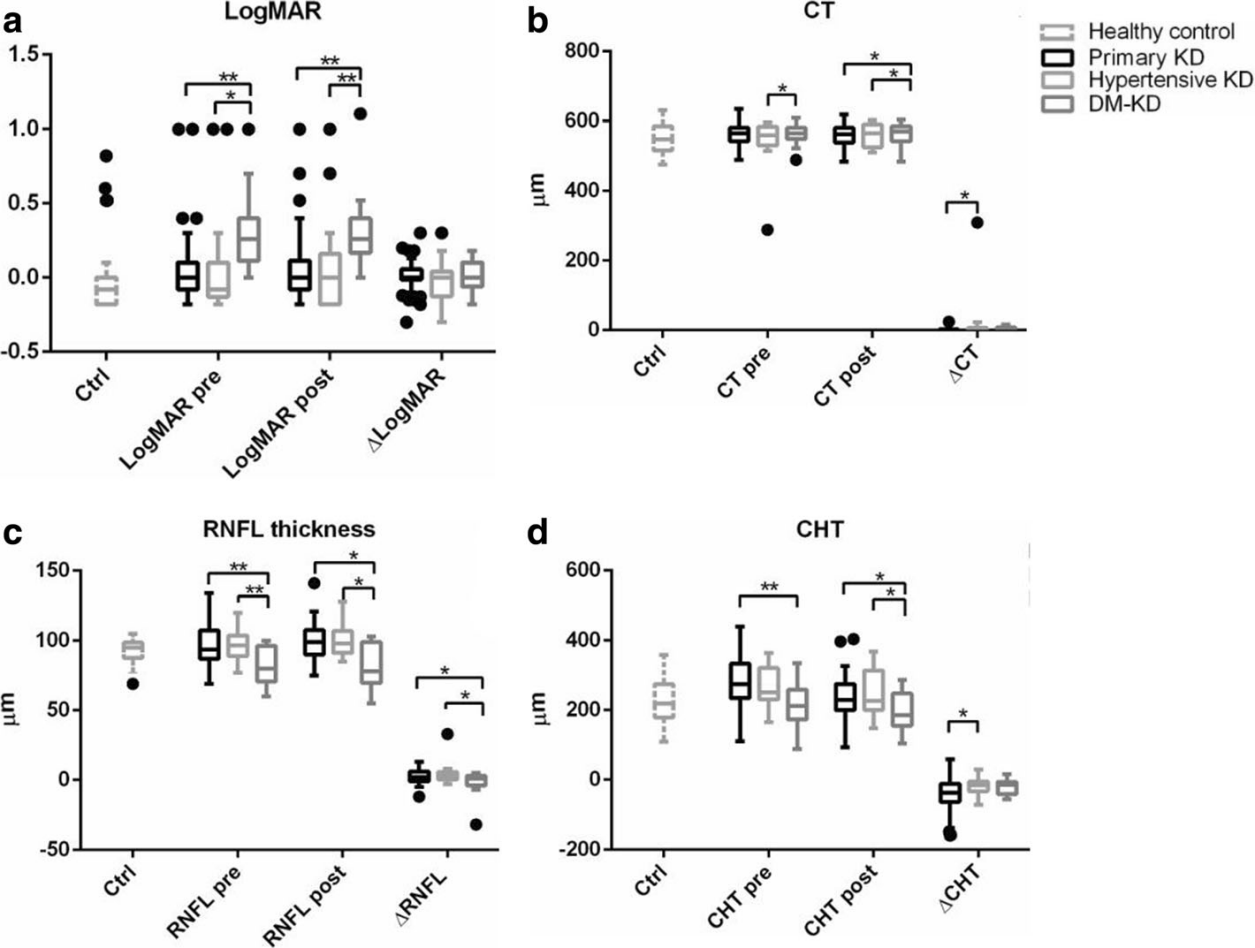
Comparison of the logMAR of the BCVA, central corneal thickness (CT), RNFL thickness and choroid thickness (CHT) among the primary KD, hypertensive KD, and DM-KD subgroups. **a** The logMAR of the BCVA of the DM-KD subgroup was significantly higher than that of the primary KD subgroup and the hypertensive KD subgroup before and after HD. **b** The CT of the DM-KD subgroup was significantly higher before and after HD. **c** The average RNFL thickness of the DM-KD subgroup was significantly lower than that of the other 2 subgroups both before and after HD, and a lower change of RNFL thickness of the DM-KD was found compared to the other 2 subgroups. The 3 bars are from left to right: the primary kidney disease (KD) subgroup (*n* = 27), the hypertensive KD subgroup (*n* = 9), the diabetic mellitus KD (DM-KD) subgroup (*n* = 6). **d** The CHT in the DM-KD subgroup was lower than that of the other 2 subgroups before and after HD. Δ value (change of value) was defined as the value after HD minus the value before HD. *: *P* < 0.05; **: *P* < 0.01

There were no differences in the sex, age and HD duration among the 3 subgroups. However, four parameters, logMAR of BCVA, CT, RNFL thickness and CHT, were significantly different among subgroups (Fig. 2).

In summary, primary KD, hypertension and diabetes mellitus are three conditions that affect eye examination results during HD to a certain degree. However, the different causes of CRF requiring hemodialysis tend to have the same overall trend in most parameter changes except the logMAR of the BCVA, CT, RNFL thickness and CHT. Lower BCVA, increased central corneal thickness, and decreased RNFL thickness and CHT were observed in the DM-KD patients.

## Discussion

All patients showed significant changes in dry eye parameters, RT and CHT. Different etiologies tended to exhibit similar trends in ocular parameter changes during HD.

HD is the process of clearing excess body water and metabolic waste. Therefore, body fluid volume, solute concentration, and crystal osmotic pressure decrease after HD. Our study shows that, consistent with most studies [2, 15], the TBUT and Schirmer’s test results significantly decrease during HD. Aggravation of dry eye syndromes may be the result of less body fluid and tear secretion. Although toxins are removed from patients during HD, these patients still resemble secretion-deficient dry eye patients after treatment and are in need of ophthalmic artificial tear replacement. Moreover, a positive correlation was found between the TBUT reduction and HD duration, indicating the cumulative effect of HD on the patients’ dry eye conditions. Dry eye symptoms can be observed immediately (within an hour) after a single HD session. Both the long-term and short-term effects of HD should be fully considered to relieve symptoms of dry eye.

Over the years, there have been a number of studies on HD, leading to changes in IOP. However, the conclusions of different scholars vary. Overall, the factors associated with post-HD IOP change are mainly measurement techniques and the anterior chamber angle. Findings obtained using the Goldmann tonometer tended to find reductions; the use of NCT resulted in opposite findings in that increased IOP is more likely to take place in a narrow and obstructive anterior chamber angle and tends to decline at an opposite angle [2, 9–11, 16, 17]. Our study suggested that the IOP has no significant change after HD regardless of the etiology of the ESRD. A negative correlation was observed between the changes in the IOP and diastolic pressure. IOP might be related not only to measurement method and anterior chamber angle, but also to blood pressure.

Early researchers believed that after HD, the ACD would either decline [2] or would not change [16]. In our study, the ACD significantly declined after HD. With the reductions in body fluid volume and osmotic pressure caused by HD, the amount of aqueous humor declined. Moreover, because this decrease is mainly due to changes in the crystal osmotic pressure rather than the colloid osmotic pressure, the latter increases after HD, and it can result in the aqueous humor flowing into the blood via the anterior chamber angle trabecular meshwork. This mechanism also contributes to reduced intraocular pressure. However, in patients with narrow or obstructive angles, this process is hampered, and due to the decline in the plasma crystal osmotic pressure, body fluid moves along the concentration gradient into the anterior chamber; this process may lead to increased IOP and even acute angle-closure glaucoma [17]. Based on this conclusion, we should consider IOP, gonioscopy, and visual field tests for HD patients. For those with a high risk of anterior chamber obstruction, more detailed follow-ups should be conducted to determine any possible existence of glaucoma. These patients are classified as high risk patients, and preventative measures should be taken in the hemodialysis unit.

During HD, retinal thickness tended to increase in different locations and different layers of the retina, including the RNFL. Different etiologies did not affect the degree of change in these parameters. However, previous research found no statistically significant differences before and after HD in terms of the macular thickness [13, 18], the thickness of the surrounding macular areas [13], the macular volume [12] or the layer of retinal ganglion cells [11, 12]. Our conclusion is not yet supported by other studies, so we propose the following hypothesis: HD reduces the plasma crystal osmotic pressure such that the liquid goes into the layers of the retina along the concentration gradient, thickening the retina and leading to edema. The specific mechanisms of our hypothesis await verification from follow-up studies.

Many studies have shown that the subfoveal, nasal, temporal and average choroidal thicknesses can be significantly reduced by HD [13, 19]. Our conclusion is the same as those of previous studies in that the average CHT significantly decreased after HD; this was especially the case in patients with DM, in whom the CHT was significantly smaller both before and after HD. The CHT change was positively related to the TBUT before HD because HD removes excess body liquid and reduces the blood volume. Thus, the choroidal vascular layer significantly “shrinks,” and the choroidal thickness is reduced. These changes are associated with certain indicators that reflect the body fluid volume. Earlier research found that the peak systolic flow velocity (PSV) and end diastolic flow velocity (EDV) both significantly decline in the temporal posterior ciliary artery (TPCA) and the central retinal artery (CRA) [20] after HD. Combined with the findings of our study, we suggest that optic blood vessels can be considered to provide relatively insufficient blood supply after hemodialysis. Therefore, in this phase, clinical physicians should pay special attention to the prevention and treatment of ocular ischemic diseases.

No obvious change in BCVA occurred after HD. Moreover, no change in vision was related to the etiology of ESRD, leading to the conclusion that HD cannot directly affect vision. A previous study suggested that HD can improve BCVA [21], although some scholars hold the opposite view, particuarly for patients with DM-KD [10]. Similarly, we found that visual acuity before and after HD in patients with DM was significantly worse than in patients with other etiologies. Diabetes may be an important factor leading to reduced visual acuity because diabetes can cause and aggravate lens turbidity.

The central CT and endothelial number and form remained unchanged after HD, and the subgroup analysis showed that CT during HD in the DM-KD subgroup was significantly greater than that in the other two subgroups. However, previous studies have found that the CT was significantly reduced [2, 5, 11, 13] or remained unchanged [12, 22] after HD; this phenomenon is related to dehydration but is not correlated with DM. Our study differed in that we first used the Oculus Pentacam Anterior Segment Analyzer to measure the CT, and we recorded the corneal vertex thickness (Pachy apex) as the central CT. We used this approach because the Pentacam measurement principle is based on using the point at the Pachy apex as a benchmark, and the remaining points are obtained relative to this point. The different conclusions between previous studies and our present study require further research.

HD failed to change the spherical and cylinder power in our study, as Çalişkan et al. [16] concluded. However, the HD procedure significantly changed the average lens thickness and density. We believe that the process of HD partially dehydrated lens so that after HD, the average thickness of the lens decreased, and the relative density increased. Although the lens thickness change was highly significant, but the change was small, just about 2% in lens thickness. As mentioned above, the BCVA of the DM-KD subgroup was significantly worse than that of the other two subgroups. Therefore, in patients with CRF, particularly those with diabetic nephropathy, early screening and treatment for vision loss should be conducted.

## Conclusions

In conclusion, renal failure patients undergoing HD may be at increased risk of developing vision-threatening complications, and both physicians of the hemodialysis unit and ophthalmologists should be made aware of this risk.

## Abbreviations

ACD: Anterior chamber depth
AVG: Average cell size
BCVA: Best corrected visual acuity
CD: Endothelial cell density
CHT: Choroidal thickness
CSF: Central subfield
CT: Corneal thickness
CV: Cell size variation coefficient
DM: Diabetes mellitus
ESRD: End-stage renal disease
HD: Hemodialysis
IIM: Inferior inner macula
IOM: Inferior outer macula
IOP: Intraocular pressure
KD: Kidney disease
logMAR: Logarithmic minimum angle of resolution
LT: Lens thickness
NIM: Nasal inner macula
NOM: Nasal outer macula
RNFL: Retinal nerve fiber layer
SIM: Superior inner macula
SOM: Superior outer macula
TBUT: Tear break-up time
TIM: Temporal inner macula
TOM: Temporal outer macula
